# Supplementary material for: Predicting firm creation in rural Texas: A multi-model machine learning approach to a complex policy problem
Source: PLoS One. 2023 Jun 23;18(6):e0287217. doi: 10.1371/journal.pone.0287217 (PMC10289456; doi:10.1371/journal.pone.0287217)

# Appendices

## Appendix A: Descriptive Statistics of All Variables

| Variable | n | Mean | Sd | Min | Max | Range | Se |
| --- | --- | --- | --- | --- | --- | --- | --- |
| Year | 2711 | NaN | NA | Inf | -Inf | -Inf | NA |
| Play | 2711 | NaN | NA | Inf | -Inf | -Inf | NA |
| Population | 2711 | 107587 | 380268 | 86 | 4680045 | 4679959 | 7303 |
| Percent_industry_farming | 2711 | 0.32 | 1.17 | 0 | 12.26 | 12.26 | 0.02 |
| Percent_industry_extraction | 2711 | 5.6 | 9.47 | 0 | 56.46 | 56.46 | 0.18 |
| Percent_industry_recreational | 2711 | 0.53 | 0.8 | 0 | 8.74 | 8.74 | 0.02 |
| Percent_industry_oil_gas | 2711 | 0.76 | 2.29 | 0 | 30 | 30 | 0.04 |
| Percent_industry_edu_prime_secondary | 2711 | 0.15 | 0.38 | 0 | 4.99 | 4.99 | 0.01 |
| Percent_industry_Manufacturing | 2711 | 6.12 | 8.91 | 0 | 53.07 | 53.07 | 0.17 |
| Percent_industry_edu_com_college | 2711 | 0 | 0 | 0 | 0.08 | 0.08 | 0 |
| Percent_industry_Healthcare | 2711 | 1.47 | 1.63 | 0 | 17.98 | 17.98 | 0.03 |
| Percent_industry_coal | 2711 | 0 | 0.12 | 0 | 5.06 | 5.06 | 0 |
| Percent_child_elder_care | 2711 | 0.85 | 5.06 | 0 | 76.58 | 76.58 | 0.1 |
| Business_density | 2711 | 27 | 72 | 2 | 1119 | 1117 | 1 |
| Industry_diversity | 2711 | 1.91 | 0.57 | 0 | 2.69 | 2.69 | 0.01 |
| Newfirms | 2695 | 418.4 | 2009.76 | 1 | 40172 | 40171 | 38.71 |
| Failedfirms | 2695 | 4.26 | 31.28 | 0 | 651 | 651 | 0.6 |
| Total_oil | 2711 | 3532015 | 11108796 | 0 | 151537156 | 151537156 | 213355 |
| Total_gas_boe | 2711 | 5827573 | 14380496 | 0 | 153312408 | 153312408 | 276191 |
| Solar_installations | 2711 | 3.69 | 20.44 | 0 | 320 | 320 | 0.39 |
| Wind_capacity | 2711 | 2449 | 31012 | 0 | 894250 | 894250 | 596 |
| Rural_per_omb | 2517 | NaN | NA | Inf | -Inf | -Inf | NA |
| Per_point_diff_2020 | 2711 | 52.67 | 19.94 | 0.49 | 91.64 | 91.15 | 0.38 |
| Percent_age_25_44 | 2711 | 0.34 | 0.06 | 0.15 | 0.52 | 0.37 | 0 |
| Percent_age_65 | 2711 | 0.2 | 0.06 | 0.05 | 0.41 | 0.36 | 0 |
| Percent_poverty | 2711 | 0.17 | 0.06 | 0.01 | 0.49 | 0.48 | 0 |
| Percent_residence_born | 2711 | 0.72 | 0.09 | 0.42 | 0.98 | 0.56 | 0 |
| Percent_edu_college | 2711 | 0.16 | 0.07 | 0.02 | 0.49 | 0.47 | 0 |
| Ethnic_diversity | 2711 | 1.17 | 0.45 | 0 | 2.06 | 2.06 | 0.01 |
| Income_inequality | 2711 | 0.45 | 0.04 | 0.34 | 0.63 | 0.29 | 0 |
| Percent_insured | 2711 | 0.96 | 0.06 | 0.62 | 1 | 0.38 | 0 |
| Percent_broadband | 2463 | 0.96 | 0.06 | 0.64 | 1 | 0.36 | 0 |
| Resilience | 2646 | 0.67 | 0.18 | 0.37 | 1.31 | 0.94 | 0 |
| Land_area | 2646 | 1022 | 657 | 129 | 6193 | 6064 | 13 |
| Distance_250k | 2646 | 76.02 | 47.41 | 0 | 223.35 | 223.35 | 0.92 |
| Natural_amenity | 2646 | 1.28 | 1.25 | -1.01 | 5.93 | 6.94 | 0.02 |
| Unemployment_rate | 2711 | 5.78 | 2.26 | 1.8 | 18.5 | 16.7 | 0.04 |
| Percent_self_employment | 2711 | 0.25 | 0.07 | 0.03 | 0.5 | 0.47 | 0 |
| Total_emp | 2711 | 63409 | 257804 | 68 | 3218399 | 3218331 | 4951 |
| Social_capital | 2711 | -0.44 | 1.17 | -2.95 | 7.16 | 10.11 | 0.02 |
| Patents | 2711 | 23.74 | 131.84 | 0 | 1429 | 1429 | 2.53 |
| Newfirms_prevyear | 2689 | 350 | 1595 | 1 | 27029 | 27028 | 31 |
| Banks | 2693 | 27.63 | 89.87 | 1 | 1079 | 1078 | 1.73 |
| Deposits_thousands | 2693 | 2645757 | 14802403 | 10128 | 211300891 | 211290763 | 285242 |
| County_gdp | 2711 | 5912664 | 27103441 | 31652 | 387731505 | 387699853 | 520547 |
| Protestant_ethic | 2711 | 46.95 | 339.12 | 0 | 4550 | 4550 | 6.51 |
| Fed_fund_rate | 2711 | 0.53 | 0.67 | 0.09 | 1.93 | 1.84 | 0.01 |
| Percent_mobility_in_mig | 2711 | 0.08 | 0.03 | 0 | 0.24 | 0.24 | 0 |
| Percent_mobility_out_mig | 2711 | 0.08 | 0.06 | 0 | 1.87 | 1.87 | 0 |
| Population_density | 2646 | 116 | 330 | 0 | 2989 | 2989 | 6 |

## Appendix B: Variable appearance across all models

| **SlNo** | **Variables** | **Linear Model** | **Forward Subset Selection** | **Backward Subset Selection** | **Lasso Regression** | **Random Forest MSE** | **Random Forest RSS** | **XG Boost** | **Total Appearances** |
| --- | --- | --- | --- | --- | --- | --- | --- | --- | --- |
| 1 | population | 1 | 1 | 0 | 1 | 1 | 1 | 1 | 6 |
| 2 | newfirms_prevyear | 1 | 1 | 0 | 1 | 1 | 1 | 1 | 6 |
| 3 | total_oil | 1 | 0 | 0 | 1 | 1 | 1 | 1 | 5 |
| 4 | banks | 0 | 1 | 1 | 0 | 1 | 1 | 1 | 5 |
| 5 | percent_mobility_in_mig | 0 | 1 | 1 | 0 | 1 | 1 | 1 | 5 |
| 6 | percent_industry_healthcare | 0 | 0 | 0 | 1 | 1 | 1 | 1 | 4 |
| 7 | percent_child_elder_care | 0 | 1 | 0 | 0 | 1 | 1 | 1 | 4 |
| 8 | failedfirms | 0 | 1 | 0 | 0 | 1 | 1 | 1 | 4 |
| 9 | wind_capacity | 0 | 0 | 0 | 1 | 1 | 1 | 1 | 4 |
| 10 | percent_age_25_44 | 0 | 0 | 0 | 1 | 1 | 1 | 1 | 4 |
| 11 | percent_age_65 | 0 | 0 | 0 | 1 | 1 | 1 | 1 | 4 |
| 12 | ethnic_diversity | 0 | 0 | 0 | 1 | 1 | 1 | 1 | 4 |
| 13 | unemployment_rate | 0 | 0 | 0 | 1 | 1 | 1 | 1 | 4 |
| 14 | total_emp | 1 | 0 | 0 | 0 | 1 | 1 | 1 | 4 |
| 15 | social_capital | 0 | 0 | 0 | 1 | 1 | 1 | 1 | 4 |
| 16 | county_gdp | 1 | 0 | 0 | 0 | 1 | 1 | 1 | 4 |
| 17 | fed_fund_rate | 1 | 0 | 0 | 0 | 1 | 1 | 1 | 4 |
| 18 | year_2011 | 1 | 1 | 1 | 1 | 0 | 0 | 0 | 4 |
| 19 | year_2013 | 1 | 1 | 1 | 1 | 0 | 0 | 0 | 4 |
| 20 | year_2014 | 0 | 1 | 1 | 1 | 0 | 0 | 1 | 4 |
| 21 | year_2016 | 0 | 1 | 1 | 1 | 0 | 0 | 1 | 4 |
| 22 | percent_industry_farming | 0 | 0 | 0 | 0 | 1 | 1 | 1 | 3 |
| 23 | percent_industry_extraction | 0 | 0 | 0 | 0 | 1 | 1 | 1 | 3 |
| 24 | percent_industry_recreational | 0 | 0 | 0 | 0 | 1 | 1 | 1 | 3 |
| 25 | percent_industry_edu_prime_secondary | 0 | 0 | 0 | 0 | 1 | 1 | 1 | 3 |
| 26 | percent_industry_manufacturing | 0 | 0 | 0 | 0 | 1 | 1 | 1 | 3 |
| 27 | business_density | 0 | 0 | 0 | 0 | 1 | 1 | 1 | 3 |
| 28 | industry_diversity | 0 | 0 | 0 | 0 | 1 | 1 | 1 | 3 |
| 29 | total_gas_boe | 0 | 0 | 0 | 0 | 1 | 1 | 1 | 3 |
| 30 | solar_installations | 0 | 0 | 0 | 0 | 1 | 1 | 1 | 3 |
| 31 | per_point_diff_2020 | 0 | 0 | 0 | 0 | 1 | 1 | 1 | 3 |
| 32 | percent_residence_born | 0 | 0 | 0 | 0 | 1 | 1 | 1 | 3 |
| 33 | percent_edu_college | 0 | 0 | 0 | 0 | 1 | 1 | 1 | 3 |
| 34 | income_inequality | 0 | 1 | 0 | 0 | 1 | 1 | 0 | 3 |
| 35 | percent_broadband | 0 | 0 | 0 | 0 | 1 | 1 | 1 | 3 |
| 36 | resilience | 0 | 0 | 0 | 0 | 1 | 1 | 1 | 3 |
| 37 | land_area | 0 | 0 | 0 | 0 | 1 | 1 | 1 | 3 |
| 38 | distance_250k | 0 | 0 | 0 | 0 | 1 | 1 | 1 | 3 |
| 39 | natural_amenity | 0 | 0 | 0 | 0 | 1 | 1 | 1 | 3 |
| 40 | percent_self_employment | 0 | 0 | 0 | 0 | 1 | 1 | 1 | 3 |
| 41 | patents | 0 | 0 | 0 | 0 | 1 | 1 | 1 | 3 |
| 42 | deposits_thousands | 0 | 0 | 0 | 0 | 1 | 1 | 1 | 3 |
| 43 | percent_mobility_out_mig | 0 | 0 | 0 | 1 | 1 | 1 | 0 | 3 |
| 44 | population_density | 0 | 0 | 0 | 0 | 1 | 1 | 1 | 3 |
| 45 | year_2010 | 1 | 1 | 1 | 0 | 0 | 0 | 0 | 3 |
| 46 | year_2012 | 0 | 0 | 1 | 1 | 0 | 0 | 1 | 3 |
| 47 | percent_industry_oil_gas | 0 | 0 | 0 | 0 | 1 | 1 | 0 | 2 |
| 48 | percent_industry_edu_com_college | 0 | 0 | 0 | 0 | 1 | 1 | 0 | 2 |
| 49 | percent_industry_coal | 0 | 0 | 0 | 0 | 1 | 1 | 0 | 2 |
| 50 | percent_poverty | 0 | 0 | 0 | 0 | 1 | 1 | 0 | 2 |
| 51 | percent_insured | 0 | 0 | 0 | 0 | 1 | 1 | 0 | 2 |
| 52 | protestant_ethic | 0 | 0 | 0 | 0 | 1 | 1 | 0 | 2 |
| 53 | year_2009 | 1 | 0 | 0 | 1 | 0 | 0 | 0 | 2 |
| 54 | year_2015 | 1 | 0 | 1 | 0 | 0 | 0 | 0 | 2 |
| 55 | play_Eagle Ford | 0 | 1 | 1 | 0 | 0 | 0 | 0 | 2 |
| 56 | year_2017 | 0 | 0 | 1 | 0 | 0 | 0 | 0 | 1 |
| 57 | play_Granite Wash | 0 | 1 | 0 | 0 | 0 | 0 | 0 | 1 |
| 58 | (Intercept) | 0 | 0 | 0 | 0 | 0 | 0 | 0 | 0 |
| 59 | year_2018 | 0 | 0 | 0 | 0 | 0 | 0 | 0 | 0 |
| 60 | play_Haynesville | 0 | 0 | 0 | 0 | 0 | 0 | 0 | 0 |
| 61 | play_None | 0 | 0 | 0 | 0 | 0 | 0 | 0 | 0 |
| 62 | play_Permian | 0 | 0 | 0 | 0 | 0 | 0 | 0 | 0 |

## Appendix C: Hyperparameter tuning plots

C.1. In our lasso regression model, we obtained the optimal lambda for minimizing test MSE through 10-fold cross validation. The plot of test MSE by lambda value is below.


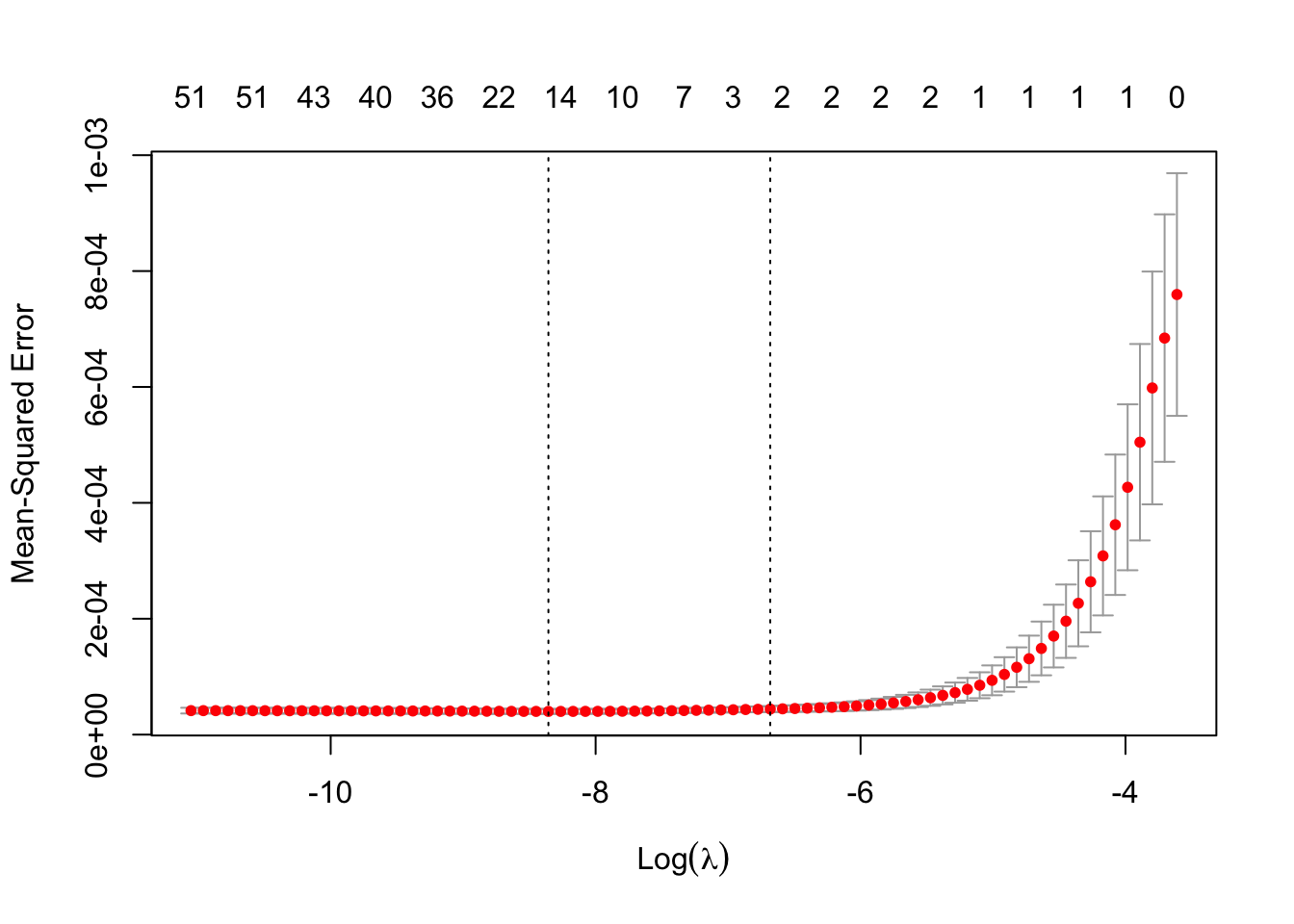


C.2. In our random forest model, we selected the optimal mtry value (the number of variables to randomly sample as candidates at each split) to minimize the OOB error rate. The tuning plot is below:


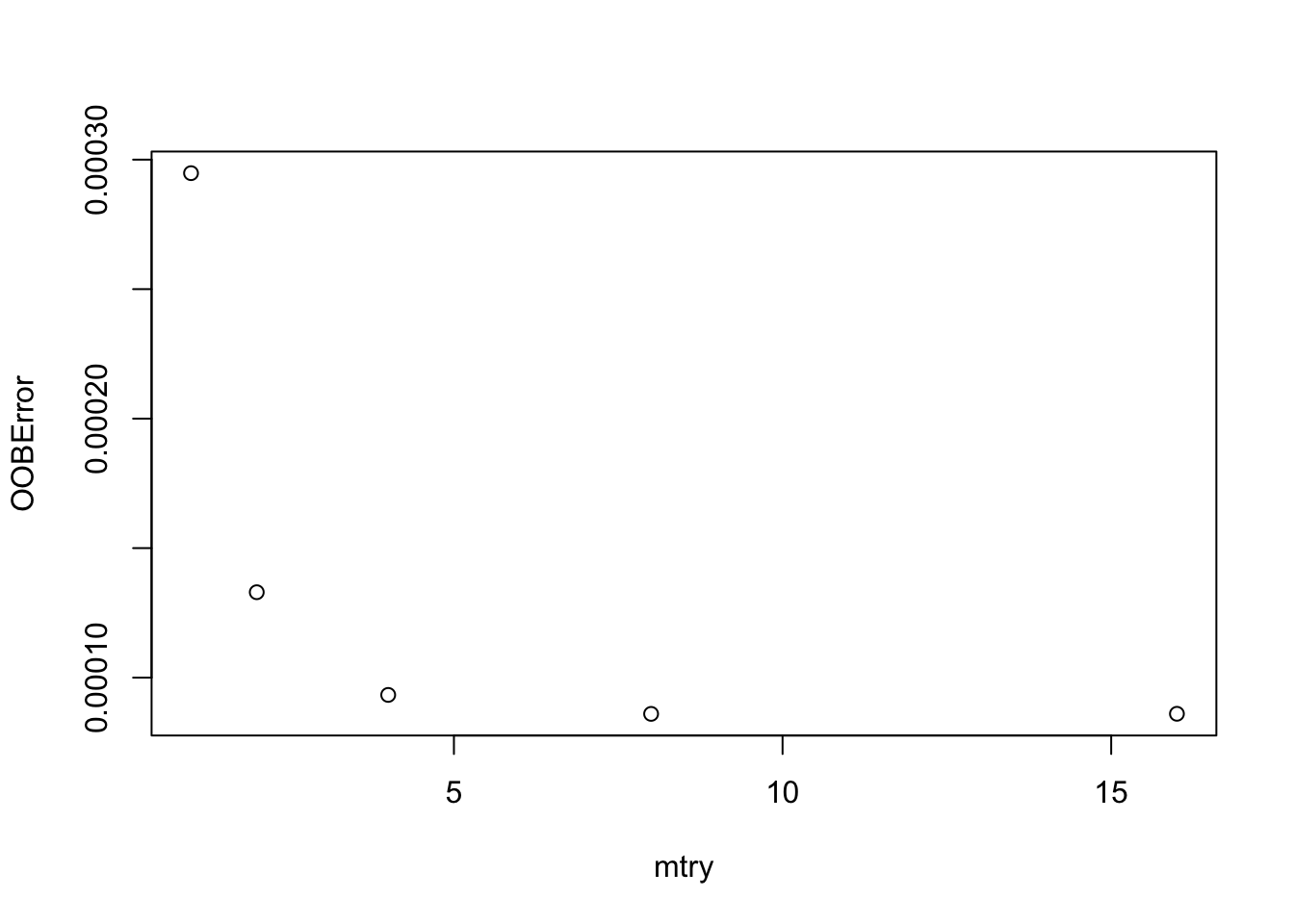

Supplement: S1 Appendix — Appendix A: Descriptive Statistics of All Variables. Appendix B: Variable appearance across all models. Appendix C: Hyperparameter tuning plots. (DOCX) [file pone.0287217.s001.docx]
