# Supplementary material for: Predicting firm creation in rural Texas: A multi-model machine learning approach to a complex policy problem
Source: PLoS One. 2023 Jun 23;18(6):e0287217. doi: 10.1371/journal.pone.0287217 (PMC10289456; doi:10.1371/journal.pone.0287217)
Supplement: S1 File — (ZIP) [file pone.0287217.s002.zip › S2 - Replication Data/newfirms_statmodels_v5_rural.html]

New firms models with time series data: Rural only


# New firms models with time series data: Rural only

#### 

#### 3/14/2023

Below is a descriptive summary table for all the variables used in the model.

|  | vars | n | mean | sd | min | max | range | se |
| --- | --- | --- | --- | --- | --- | --- | --- | --- |
| year | 1 | 2711 | NaN | NA | Inf | -Inf | -Inf | NA |
| play | 2 | 2711 | NaN | NA | Inf | -Inf | -Inf | NA |
| population | 3 | 2711 | 107587.16 | 380268.04 | 86.00 | 4680045.00 | 4679959.00 | 7303.40 |
| percent\_industry\_farming | 4 | 2711 | 0.32 | 1.17 | 0.00 | 12.26 | 12.26 | 0.02 |
| percent\_industry\_extraction | 5 | 2711 | 5.60 | 9.47 | 0.00 | 56.46 | 56.46 | 0.18 |
| percent\_industry\_recreational | 6 | 2711 | 0.53 | 0.80 | 0.00 | 8.74 | 8.74 | 0.02 |
| percent\_industry\_oil\_gas | 7 | 2711 | 0.76 | 2.29 | 0.00 | 30.00 | 30.00 | 0.04 |
| percent\_industry\_edu\_prime\_secondary | 8 | 2711 | 0.15 | 0.38 | 0.00 | 4.99 | 4.99 | 0.01 |
| percent\_industry\_manufacturing | 9 | 2711 | 6.12 | 8.91 | 0.00 | 53.07 | 53.07 | 0.17 |
| percent\_industry\_edu\_com\_college | 10 | 2711 | 0.00 | 0.00 | 0.00 | 0.08 | 0.08 | 0.00 |
| percent\_industry\_healthcare | 11 | 2711 | 1.47 | 1.63 | 0.00 | 17.98 | 17.98 | 0.03 |
| percent\_industry\_coal | 12 | 2711 | 0.00 | 0.12 | 0.00 | 5.06 | 5.06 | 0.00 |
| percent\_child\_elder\_care | 13 | 2711 | 0.85 | 5.06 | 0.00 | 76.58 | 76.58 | 0.10 |
| business\_density | 14 | 2711 | 26.76 | 72.18 | 1.73 | 1119.02 | 1117.29 | 1.39 |
| industry\_diversity | 15 | 2711 | 1.91 | 0.57 | 0.00 | 2.69 | 2.69 | 0.01 |
| newfirms | 16 | 2695 | 418.40 | 2009.76 | 1.00 | 40172.00 | 40171.00 | 38.71 |
| failedfirms | 17 | 2695 | 4.26 | 31.28 | 0.00 | 651.00 | 651.00 | 0.60 |
| total\_oil | 18 | 2711 | 3532014.99 | 11108796.38 | 0.00 | 151537156.00 | 151537156.00 | 213354.72 |
| total\_gas\_boe | 19 | 2711 | 5827573.15 | 14380496.10 | 0.00 | 153312408.00 | 153312408.00 | 276190.74 |
| solar\_installations | 20 | 2711 | 3.69 | 20.44 | 0.00 | 320.00 | 320.00 | 0.39 |
| wind\_capacity | 21 | 2711 | 2448.78 | 31011.58 | 0.00 | 894249.50 | 894249.50 | 595.61 |
| rural\_per\_omb | 22 | 2517 | NaN | NA | Inf | -Inf | -Inf | NA |
| per\_point\_diff\_2020 | 23 | 2711 | 52.67 | 19.94 | 0.49 | 91.64 | 91.15 | 0.38 |
| percent\_age\_25\_44 | 24 | 2711 | 0.34 | 0.06 | 0.15 | 0.52 | 0.37 | 0.00 |
| percent\_age\_65 | 25 | 2711 | 0.20 | 0.06 | 0.05 | 0.41 | 0.36 | 0.00 |
| percent\_poverty | 26 | 2711 | 0.17 | 0.06 | 0.01 | 0.49 | 0.48 | 0.00 |
| percent\_residence\_born | 27 | 2711 | 0.72 | 0.09 | 0.42 | 0.98 | 0.56 | 0.00 |
| percent\_edu\_college | 28 | 2711 | 0.16 | 0.07 | 0.02 | 0.49 | 0.47 | 0.00 |
| ethnic\_diversity | 29 | 2711 | 1.17 | 0.45 | 0.00 | 2.06 | 2.06 | 0.01 |
| income\_inequality | 30 | 2711 | 0.45 | 0.04 | 0.34 | 0.63 | 0.29 | 0.00 |
| percent\_insured | 31 | 2711 | 0.96 | 0.06 | 0.62 | 1.00 | 0.38 | 0.00 |
| percent\_broadband | 32 | 2463 | 0.96 | 0.06 | 0.64 | 1.00 | 0.36 | 0.00 |
| resilience | 33 | 2646 | 0.67 | 0.18 | 0.37 | 1.31 | 0.94 | 0.00 |
| land\_area | 34 | 2646 | 1021.97 | 657.13 | 128.79 | 6192.61 | 6063.82 | 12.77 |
| distance\_250k | 35 | 2646 | 76.02 | 47.41 | 0.00 | 223.35 | 223.35 | 0.92 |
| natural\_amenity | 36 | 2646 | 1.28 | 1.25 | -1.01 | 5.93 | 6.94 | 0.02 |
| unemployment\_rate | 37 | 2711 | 5.78 | 2.26 | 1.80 | 18.50 | 16.70 | 0.04 |
| percent\_self\_employment | 38 | 2711 | 0.25 | 0.07 | 0.03 | 0.50 | 0.47 | 0.00 |
| total\_emp | 39 | 2711 | 63408.65 | 257803.52 | 68.00 | 3218399.00 | 3218331.00 | 4951.36 |
| social\_capital | 40 | 2711 | -0.44 | 1.17 | -2.95 | 7.16 | 10.11 | 0.02 |
| patents | 41 | 2711 | 23.74 | 131.84 | 0.00 | 1429.00 | 1429.00 | 2.53 |
| newfirms\_prevyear | 42 | 2689 | 350.18 | 1594.60 | 1.00 | 27029.00 | 27028.00 | 30.75 |
| banks | 43 | 2693 | 27.63 | 89.87 | 1.00 | 1079.00 | 1078.00 | 1.73 |
| deposits\_thousands | 44 | 2693 | 2645757.39 | 14802403.39 | 10128.00 | 211300891.00 | 211290763.00 | 285242.38 |
| county\_gdp | 45 | 2711 | 5912664.04 | 27103441.34 | 31652.00 | 387731505.00 | 387699853.00 | 520546.68 |
| protestant\_ethic | 46 | 2711 | 46.95 | 339.12 | 0.00 | 4550.00 | 4550.00 | 6.51 |
| fed\_fund\_rate | 47 | 2711 | 0.53 | 0.67 | 0.09 | 1.93 | 1.84 | 0.01 |
| percent\_mobility\_in\_mig | 48 | 2711 | 0.08 | 0.03 | 0.00 | 0.24 | 0.24 | 0.00 |
| percent\_mobility\_out\_mig | 49 | 2711 | 0.08 | 0.06 | 0.00 | 1.87 | 1.87 | 0.00 |
| population\_density | 50 | 2646 | 115.89 | 330.39 | 0.13 | 2989.26 | 2989.13 | 6.42 |

Below is a plot of New firms registered in Texas from 2008-2018

# Linear regression model

Coefficients significant at 10% level arranged in descending order are:

|  | Variable | Coefficient | Direction |
| --- | --- | --- | --- |
| newfirms\_prevyear | newfirms\_prevyear | 0.739 | ++ |
| population | population | 0.073 | ++ |
| total\_emp | total\_emp | 0.029 | – |
| county\_gdp | county\_gdp | 0.029 | ++ |
| year\_2009 | year\_2009 | 0.005 | – |
| year\_2013 | year\_2013 | 0.005 | – |
| year\_2015 | year\_2015 | 0.004 | – |
| year\_2010 | year\_2010 | 0.003 | – |
| fed\_fund\_rate | fed\_fund\_rate | 0.002 | – |
| year\_2011 | year\_2011 | 0.002 | – |
| total\_oil | total\_oil | 0.001 | – |

Test MSE for linear regresison model is 1.6764768^{-4}. 10-fold CV error for linear regresison model is 4.8893113^{-5}.

# Forward subset selection model

The coefficients for the model with the lowest CV error are:

```
## Reordering variables and trying again:
```

|  | Variable | Coefficient | Direction |
| --- | --- | --- | --- |
| newfirms\_prevyear | newfirms\_prevyear | 0.891 | ++ |
| failedfirms | failedfirms | 0.009 | ++ |
| population | population | 0.007 | ++ |
| year\_2013 | year\_2013 | 0.004 | – |
| year\_2010 | year\_2010 | 0.001 | – |
| percent\_child\_elder\_care | percent\_child\_elder\_care | 0.000 | ++ |
| income\_inequality | income\_inequality | 0.000 | – |
| banks | banks | 0.000 | – |
| percent\_mobility\_in\_mig | percent\_mobility\_in\_mig | 0.000 | ++ |
| year\_2011 | year\_2011 | 0.000 | ++ |
| year\_2014 | year\_2014 | 0.000 | ++ |
| year\_2016 | year\_2016 | 0.000 | – |
| `play_Eagle Ford` | `play_Eagle Ford` | 0.000 | – |
| `play_Granite Wash` | `play_Granite Wash` | 0.000 | – |

The lowest cross-validation error in forward subset selection is for a model with 14 variables, and the corresponding 10-fold CV error is 0.0068186.

# Backward subset selection model

```
## Reordering variables and trying again:
```

|  | Variable | Coefficient | Direction |
| --- | --- | --- | --- |
| banks | banks | 0.014 | ++ |
| year\_2017 | year\_2017 | 0.012 | ++ |
| year\_2010 | year\_2010 | 0.008 | – |
| year\_2011 | year\_2011 | 0.006 | – |
| year\_2016 | year\_2016 | 0.006 | ++ |
| percent\_mobility\_in\_mig | percent\_mobility\_in\_mig | 0.004 | – |
| `play_Eagle Ford` | `play_Eagle Ford` | 0.003 | ++ |
| year\_2013 | year\_2013 | 0.002 | – |
| year\_2014 | year\_2014 | 0.002 | ++ |
| year\_2015 | year\_2015 | 0.002 | ++ |
| year\_2012 | year\_2012 | 0.001 | – |

The lowest cross-validation error in backward subset selection is for a model with 11 variables, and the corresponding 10-fold CV error is 0.0068328.

# Lasso regression

Selected coefficients arranged in descending order are:

|  | Variable | Coefficient | Direction |
| --- | --- | --- | --- |
| newfirms\_prevyear | newfirms\_prevyear | 0.777 | ++ |
| population | population | 0.041 | ++ |
| wind\_capacity | wind\_capacity | 0.016 | ++ |
| year\_2012 | year\_2012 | 0.003 | ++ |
| year\_2014 | year\_2014 | 0.002 | ++ |
| year\_2016 | year\_2016 | 0.002 | ++ |
| year\_2009 | year\_2009 | 0.001 | – |
| year\_2011 | year\_2011 | 0.001 | ++ |
| year\_2013 | year\_2013 | 0.001 | – |
| percent\_industry\_healthcare | percent\_industry\_healthcare | 0.000 | – |
| total\_oil | total\_oil | 0.000 | – |
| percent\_age\_25\_44 | percent\_age\_25\_44 | 0.000 | – |
| percent\_age\_65 | percent\_age\_65 | 0.000 | ++ |
| ethnic\_diversity | ethnic\_diversity | 0.000 | ++ |
| unemployment\_rate | unemployment\_rate | 0.000 | – |
| social\_capital | social\_capital | 0.000 | ++ |
| percent\_mobility\_out\_mig | percent\_mobility\_out\_mig | 0.000 | – |

Test MSE for lasso regression model is 0.0445823. 10-fold CV error for lasso regresison model is 0.0495214.

The optimal lambda for minimizing test MSE was obtained through 10-fold cross validation. The plot of test MSE by lambda value is below.

# Random forest

The top 15 variables in terms of contribution to percentage increase in MSE are:

|  | Variable | %IncMSE |
| --- | --- | --- |
| newfirms\_prevyear | newfirms\_prevyear | 16.6436143 |
| population\_density | population\_density | 13.3050607 |
| deposits\_thousands | deposits\_thousands | 11.6166912 |
| population | population | 9.3382233 |
| total\_emp | total\_emp | 9.3017303 |
| business\_density | business\_density | 8.5965867 |
| percent\_age\_25\_44 | percent\_age\_25\_44 | 8.3813034 |
| year | year | 8.3376940 |
| banks | banks | 7.9868556 |
| percent\_self\_employment | percent\_self\_employment | 7.7893071 |
| ethnic\_diversity | ethnic\_diversity | 7.2929395 |
| industry\_diversity | industry\_diversity | 7.2561963 |
| county\_gdp | county\_gdp | 7.2244431 |
| percent\_industry\_manufacturing | percent\_industry\_manufacturing | 6.7999729 |
| percent\_edu\_college | percent\_edu\_college | 6.7912959 |
| percent\_industry\_healthcare | percent\_industry\_healthcare | 6.7669616 |
| distance\_250k | distance\_250k | 6.6982201 |
| play | play | 5.6801336 |
| protestant\_ethic | protestant\_ethic | 5.6417392 |
| total\_gas\_boe | total\_gas\_boe | 5.6408628 |
| unemployment\_rate | unemployment\_rate | 5.2149217 |
| percent\_industry\_extraction | percent\_industry\_extraction | 5.0097240 |
| total\_oil | total\_oil | 4.9520532 |
| income\_inequality | income\_inequality | 4.6084806 |
| percent\_child\_elder\_care | percent\_child\_elder\_care | 4.3500618 |
| percent\_industry\_recreational | percent\_industry\_recreational | 4.3024492 |
| land\_area | land\_area | 4.2150510 |
| resilience | resilience | 4.1877893 |
| percent\_broadband | percent\_broadband | 4.0594191 |
| percent\_poverty | percent\_poverty | 4.0158570 |
| social\_capital | social\_capital | 3.8767456 |
| natural\_amenity | natural\_amenity | 3.7787712 |
| percent\_age\_65 | percent\_age\_65 | 3.6878495 |
| percent\_mobility\_in\_mig | percent\_mobility\_in\_mig | 3.6853810 |
| percent\_residence\_born | percent\_residence\_born | 3.6162419 |
| per\_point\_diff\_2020 | per\_point\_diff\_2020 | 3.3321666 |
| percent\_industry\_edu\_prime\_secondary | percent\_industry\_edu\_prime\_secondary | 3.1900661 |
| percent\_mobility\_out\_mig | percent\_mobility\_out\_mig | 3.0581149 |
| percent\_insured | percent\_insured | 2.5490297 |
| patents | patents | 2.0854701 |
| fed\_fund\_rate | fed\_fund\_rate | 2.0028104 |
| percent\_industry\_farming | percent\_industry\_farming | 1.6580817 |
| solar\_installations | solar\_installations | 1.4931302 |
| wind\_capacity | wind\_capacity | 1.3675926 |
| percent\_industry\_oil\_gas | percent\_industry\_oil\_gas | 1.0781973 |
| failedfirms | failedfirms | 0.3491223 |
| percent\_industry\_edu\_com\_college | percent\_industry\_edu\_com\_college | 0.0000000 |
| percent\_industry\_coal | percent\_industry\_coal | 0.0000000 |

The random forest model was tuned to select the optimal mtry value (the number of variables to randomly sample as candidates at each split) to minimize the OOB error rate. The tuning plot is below:

The top 15 variables in terms of contribution to percentage increase in node purity (residual sum of squares) are:

|  | Variable | IncNodePurity |
| --- | --- | --- |
| newfirms\_prevyear | newfirms\_prevyear | 0.1844784 |
| population | population | 0.1461276 |
| population\_density | population\_density | 0.1342148 |
| total\_emp | total\_emp | 0.0746451 |
| deposits\_thousands | deposits\_thousands | 0.0715795 |
| business\_density | business\_density | 0.0588810 |
| banks | banks | 0.0483792 |
| industry\_diversity | industry\_diversity | 0.0243353 |
| percent\_industry\_healthcare | percent\_industry\_healthcare | 0.0189529 |
| county\_gdp | county\_gdp | 0.0154185 |
| percent\_industry\_manufacturing | percent\_industry\_manufacturing | 0.0138875 |
| failedfirms | failedfirms | 0.0121218 |
| percent\_industry\_recreational | percent\_industry\_recreational | 0.0111284 |
| year | year | 0.0102097 |
| land\_area | land\_area | 0.0079439 |
| distance\_250k | distance\_250k | 0.0056827 |
| total\_oil | total\_oil | 0.0055042 |
| percent\_mobility\_in\_mig | percent\_mobility\_in\_mig | 0.0045037 |
| percent\_insured | percent\_insured | 0.0043164 |
| social\_capital | social\_capital | 0.0043015 |
| percent\_edu\_college | percent\_edu\_college | 0.0040285 |
| total\_gas\_boe | total\_gas\_boe | 0.0039663 |
| percent\_mobility\_out\_mig | percent\_mobility\_out\_mig | 0.0038870 |
| natural\_amenity | natural\_amenity | 0.0037970 |
| patents | patents | 0.0037893 |
| unemployment\_rate | unemployment\_rate | 0.0037745 |
| percent\_age\_25\_44 | percent\_age\_25\_44 | 0.0037272 |
| per\_point\_diff\_2020 | per\_point\_diff\_2020 | 0.0035430 |
| ethnic\_diversity | ethnic\_diversity | 0.0034994 |
| percent\_residence\_born | percent\_residence\_born | 0.0033798 |
| percent\_self\_employment | percent\_self\_employment | 0.0032230 |
| income\_inequality | income\_inequality | 0.0030635 |
| resilience | resilience | 0.0027578 |
| percent\_age\_65 | percent\_age\_65 | 0.0026866 |
| percent\_poverty | percent\_poverty | 0.0020868 |
| protestant\_ethic | protestant\_ethic | 0.0019911 |
| percent\_industry\_extraction | percent\_industry\_extraction | 0.0018877 |
| fed\_fund\_rate | fed\_fund\_rate | 0.0018259 |
| percent\_industry\_farming | percent\_industry\_farming | 0.0018066 |
| percent\_broadband | percent\_broadband | 0.0017482 |
| play | play | 0.0015998 |
| wind\_capacity | wind\_capacity | 0.0014878 |
| percent\_child\_elder\_care | percent\_child\_elder\_care | 0.0013437 |
| percent\_industry\_oil\_gas | percent\_industry\_oil\_gas | 0.0010217 |
| percent\_industry\_edu\_prime\_secondary | percent\_industry\_edu\_prime\_secondary | 0.0006431 |
| solar\_installations | solar\_installations | 0.0000793 |
| percent\_industry\_edu\_com\_college | percent\_industry\_edu\_com\_college | 0.0000000 |
| percent\_industry\_coal | percent\_industry\_coal | 0.0000000 |

Test MSE for random forest model is 8.8331592^{-4}. 10-fold CV error for random forest model is 8.1855147^{-5}.

# Extreme gradient Boosting

```
##                                 Variable        Gain      Cover   Frequency
##  1:                           population 0.474573821 0.10947419 0.098130841
##  2:                    newfirms_prevyear 0.225091081 0.13917028 0.112149533
##  3:                   population_density 0.102518817 0.03169320 0.032710280
##  4:                          failedfirms 0.076574281 0.01165461 0.009345794
##  5:                            total_emp 0.051970760 0.07157742 0.060747664
##  6:                     business_density 0.036222101 0.02027014 0.023364486
##  7:                              patents 0.006891826 0.04477569 0.046728972
##  8:                   industry_diversity 0.005658589 0.04323203 0.042056075
##  9:                   deposits_thousands 0.004175898 0.04682103 0.037383178
## 10:                     ethnic_diversity 0.003202949 0.05786782 0.060747664
## 11:       percent_industry_manufacturing 0.002600723 0.03520502 0.028037383
## 12: percent_industry_edu_prime_secondary 0.001475818 0.02097443 0.023364486
## 13:                            year_2012 0.001414419 0.04432224 0.037383178
## 14:                           county_gdp 0.001270225 0.03772311 0.042056075
## 15:          percent_industry_healthcare 0.001049691 0.02195851 0.023364486
```

Test MSE for extreme gradient boosting model is 4.4239699^{-4}. 10-fold CV error for extreme gradient boosting model is 8.5286551^{-5}.

# Comparison of Test MSE

| Model | TestMSE |
| --- | --- |
| Linear Regression | 0.000168 |
| Forward Subset Selection | 0.000137 |
| Backward Subset Selection | 0.000114 |
| Lasso Regression | 0.044582 |
| Random Forest | 0.000883 |
| XG Boost | 0.000442 |

# Comparison of 10-fold CV error

| Model | TestMSE |
| --- | --- |
| Linear Regression | 0.000049 |
| Forward Subset Selection | 0.006819 |
| Backward Subset Selection | 0.006833 |
| Lasso Regression | 0.049521 |
| Random Forest | 0.000082 |
| XG Boost | 0.000085 |

# Variable appearance across models

| SlNo | Variables | Linear Model | Forward Subset Selection | Backward Subset Selection | Lasso Regression | Random Forest MSE | Random Forest RSS | XG Boost | Total Appearances |
| --- | --- | --- | --- | --- | --- | --- | --- | --- | --- |
| 1 | population | 1 | 1 | 0 | 1 | 1 | 1 | 1 | 6 |
| 2 | newfirms\_prevyear | 1 | 1 | 0 | 1 | 1 | 1 | 1 | 6 |
| 3 | total\_oil | 1 | 0 | 0 | 1 | 1 | 1 | 1 | 5 |
| 4 | banks | 0 | 1 | 1 | 0 | 1 | 1 | 1 | 5 |
| 5 | percent\_mobility\_in\_mig | 0 | 1 | 1 | 0 | 1 | 1 | 1 | 5 |
| 6 | percent\_industry\_healthcare | 0 | 0 | 0 | 1 | 1 | 1 | 1 | 4 |
| 7 | percent\_child\_elder\_care | 0 | 1 | 0 | 0 | 1 | 1 | 1 | 4 |
| 8 | failedfirms | 0 | 1 | 0 | 0 | 1 | 1 | 1 | 4 |
| 9 | wind\_capacity | 0 | 0 | 0 | 1 | 1 | 1 | 1 | 4 |
| 10 | percent\_age\_25\_44 | 0 | 0 | 0 | 1 | 1 | 1 | 1 | 4 |
| 11 | percent\_age\_65 | 0 | 0 | 0 | 1 | 1 | 1 | 1 | 4 |
| 12 | ethnic\_diversity | 0 | 0 | 0 | 1 | 1 | 1 | 1 | 4 |
| 13 | unemployment\_rate | 0 | 0 | 0 | 1 | 1 | 1 | 1 | 4 |
| 14 | total\_emp | 1 | 0 | 0 | 0 | 1 | 1 | 1 | 4 |
| 15 | social\_capital | 0 | 0 | 0 | 1 | 1 | 1 | 1 | 4 |
| 16 | county\_gdp | 1 | 0 | 0 | 0 | 1 | 1 | 1 | 4 |
| 17 | fed\_fund\_rate | 1 | 0 | 0 | 0 | 1 | 1 | 1 | 4 |
| 18 | year\_2011 | 1 | 1 | 1 | 1 | 0 | 0 | 0 | 4 |
| 19 | year\_2013 | 1 | 1 | 1 | 1 | 0 | 0 | 0 | 4 |
| 20 | year\_2014 | 0 | 1 | 1 | 1 | 0 | 0 | 1 | 4 |
| 21 | year\_2016 | 0 | 1 | 1 | 1 | 0 | 0 | 1 | 4 |
| 22 | percent\_industry\_farming | 0 | 0 | 0 | 0 | 1 | 1 | 1 | 3 |
| 23 | percent\_industry\_extraction | 0 | 0 | 0 | 0 | 1 | 1 | 1 | 3 |
| 24 | percent\_industry\_recreational | 0 | 0 | 0 | 0 | 1 | 1 | 1 | 3 |
| 25 | percent\_industry\_edu\_prime\_secondary | 0 | 0 | 0 | 0 | 1 | 1 | 1 | 3 |
| 26 | percent\_industry\_manufacturing | 0 | 0 | 0 | 0 | 1 | 1 | 1 | 3 |
| 27 | business\_density | 0 | 0 | 0 | 0 | 1 | 1 | 1 | 3 |
| 28 | industry\_diversity | 0 | 0 | 0 | 0 | 1 | 1 | 1 | 3 |
| 29 | total\_gas\_boe | 0 | 0 | 0 | 0 | 1 | 1 | 1 | 3 |
| 30 | solar\_installations | 0 | 0 | 0 | 0 | 1 | 1 | 1 | 3 |
| 31 | per\_point\_diff\_2020 | 0 | 0 | 0 | 0 | 1 | 1 | 1 | 3 |
| 32 | percent\_residence\_born | 0 | 0 | 0 | 0 | 1 | 1 | 1 | 3 |
| 33 | percent\_edu\_college | 0 | 0 | 0 | 0 | 1 | 1 | 1 | 3 |
| 34 | income\_inequality | 0 | 1 | 0 | 0 | 1 | 1 | 0 | 3 |
| 35 | percent\_broadband | 0 | 0 | 0 | 0 | 1 | 1 | 1 | 3 |
| 36 | resilience | 0 | 0 | 0 | 0 | 1 | 1 | 1 | 3 |
| 37 | land\_area | 0 | 0 | 0 | 0 | 1 | 1 | 1 | 3 |
| 38 | distance\_250k | 0 | 0 | 0 | 0 | 1 | 1 | 1 | 3 |
| 39 | natural\_amenity | 0 | 0 | 0 | 0 | 1 | 1 | 1 | 3 |
| 40 | percent\_self\_employment | 0 | 0 | 0 | 0 | 1 | 1 | 1 | 3 |
| 41 | patents | 0 | 0 | 0 | 0 | 1 | 1 | 1 | 3 |
| 42 | deposits\_thousands | 0 | 0 | 0 | 0 | 1 | 1 | 1 | 3 |
| 43 | percent\_mobility\_out\_mig | 0 | 0 | 0 | 1 | 1 | 1 | 0 | 3 |
| 44 | population\_density | 0 | 0 | 0 | 0 | 1 | 1 | 1 | 3 |
| 45 | year\_2010 | 1 | 1 | 1 | 0 | 0 | 0 | 0 | 3 |
| 46 | year\_2012 | 0 | 0 | 1 | 1 | 0 | 0 | 1 | 3 |
| 47 | percent\_industry\_oil\_gas | 0 | 0 | 0 | 0 | 1 | 1 | 0 | 2 |
| 48 | percent\_industry\_edu\_com\_college | 0 | 0 | 0 | 0 | 1 | 1 | 0 | 2 |
| 49 | percent\_industry\_coal | 0 | 0 | 0 | 0 | 1 | 1 | 0 | 2 |
| 50 | percent\_poverty | 0 | 0 | 0 | 0 | 1 | 1 | 0 | 2 |
| 51 | percent\_insured | 0 | 0 | 0 | 0 | 1 | 1 | 0 | 2 |
| 52 | protestant\_ethic | 0 | 0 | 0 | 0 | 1 | 1 | 0 | 2 |
| 53 | year\_2009 | 1 | 0 | 0 | 1 | 0 | 0 | 0 | 2 |
| 54 | year\_2015 | 1 | 0 | 1 | 0 | 0 | 0 | 0 | 2 |
| 55 | `play_Eagle Ford` | 0 | 1 | 1 | 0 | 0 | 0 | 0 | 2 |
| 56 | year\_2017 | 0 | 0 | 1 | 0 | 0 | 0 | 0 | 1 |
| 57 | `play_Granite Wash` | 0 | 1 | 0 | 0 | 0 | 0 | 0 | 1 |
| 58 | (Intercept) | 0 | 0 | 0 | 0 | 0 | 0 | 0 | 0 |
| 59 | year\_2018 | 0 | 0 | 0 | 0 | 0 | 0 | 0 | 0 |
| 60 | play\_Haynesville | 0 | 0 | 0 | 0 | 0 | 0 | 0 | 0 |
| 61 | play\_None | 0 | 0 | 0 | 0 | 0 | 0 | 0 | 0 |
| 62 | play\_Permian | 0 | 0 | 0 | 0 | 0 | 0 | 0 | 0 |

# Variable Ranking

These two “importance ranks” have different penalties for not appearing in a model: \* ‘importance1’ gives NAs (of variables not in the model) a value of n+1, where n = the total number of variables in the largest model.

| Variable | rank\_importance\_n |
| --- | --- |
| year\_2014 | 1 |
| year\_2016 | 2 |
| newfirms\_prevyear | 3 |
| year\_2011 | 4 |
| year\_2013 | 5 |
| population | 6 |
| year\_2012 | 7 |
| wind\_capacity | 8 |
| year\_2009 | 9 |
| percent\_industry\_healthcare | 10 |
| total\_oil | 11 |
| percent\_age\_25\_44 | 12 |
| percent\_age\_65 | 13 |
| ethnic\_diversity | 14 |
| unemployment\_rate | 15 |
| social\_capital | 16 |
| percent\_mobility\_out\_mig | 17 |
| banks | 18 |
| year\_2010 | 19 |
| percent\_mobility\_in\_mig | 20 |
| `play_Eagle Ford` | 21 |
| failedfirms | 22 |
| year\_2017 | 23 |
| percent\_child\_elder\_care | 24 |
| income\_inequality | 25 |
| year\_2015 | 26 |
| `play_Granite Wash` | 27 |
| total\_emp | 28 |
| county\_gdp | 29 |
| population\_density | 30 |
| deposits\_thousands | 31 |
| business\_density | 32 |
| industry\_diversity | 33 |
| percent\_industry\_manufacturing | 34 |
| percent\_edu\_college | 35 |
| percent\_self\_employment | 36 |
| percent\_industry\_recreational | 37 |
| distance\_250k | 38 |
| fed\_fund\_rate | 39 |
| patents | 40 |
| total\_gas\_boe | 41 |
| natural\_amenity | 42 |
| land\_area | 43 |
| resilience | 44 |
| percent\_residence\_born | 45 |
| percent\_industry\_edu\_prime\_secondary | 46 |
| percent\_industry\_extraction | 47 |
| per\_point\_diff\_2020 | 48 |
| percent\_broadband | 49 |
| percent\_industry\_farming | 50 |
| protestant\_ethic | 51 |
| percent\_insured | 52 |
| percent\_poverty | 53 |
| solar\_installations | 54 |
| percent\_industry\_oil\_gas | 55 |
| percent\_industry\_edu\_com\_college | 56 |
| percent\_industry\_coal | 57 |
| (Intercept) | 60 |
| year\_2018 | 60 |
| play\_Haynesville | 60 |
| play\_None | 60 |
| play\_Permian | 60 |

- ‘importance2’ gives a the NAs a value of m, where m+1, where m = the number the number of variables included within a specific model

| Variable | rank\_importance\_m |
| --- | --- |
| newfirms\_prevyear | 1 |
| population | 2 |
| wind\_capacity | 3 |
| year\_2012 | 4 |
| year\_2014 | 5 |
| year\_2016 | 6 |
| year\_2009 | 7 |
| year\_2011 | 8 |
| year\_2013 | 9 |
| year\_2010 | 10 |
| banks | 11 |
| year\_2017 | 12 |
| failedfirms | 13 |
| percent\_mobility\_in\_mig | 14 |
| percent\_child\_elder\_care | 15 |
| income\_inequality | 16 |
| population\_density | 17 |
| total\_emp | 18 |
| deposits\_thousands | 19 |
| business\_density | 20 |
| industry\_diversity | 21 |
| year\_2015 | 22 |
| `play_Eagle Ford` | 23 |
| (Intercept) | 26 |
| year\_2018 | 26 |
| play\_Haynesville | 26 |
| play\_None | 26 |
| play\_Permian | 26 |
| county\_gdp | 29 |
| percent\_industry\_manufacturing | 30 |
| percent\_industry\_healthcare | 31 |
| percent\_edu\_college | 32 |
| percent\_self\_employment | 33 |
| percent\_industry\_recreational | 34 |
| distance\_250k | 35 |
| protestant\_ethic | 36 |
| percent\_insured | 37 |
| patents | 38 |
| percent\_poverty | 39 |
| total\_gas\_boe | 40 |
| natural\_amenity | 41 |
| land\_area | 42 |
| resilience | 43 |
| percent\_residence\_born | 44 |
| percent\_industry\_edu\_prime\_secondary | 45 |
| fed\_fund\_rate | 46 |
| percent\_industry\_oil\_gas | 47 |
| percent\_industry\_extraction | 48 |
| per\_point\_diff\_2020 | 49 |
| percent\_industry\_edu\_com\_college | 50 |
| percent\_industry\_coal | 51 |
| percent\_broadband | 52 |
| percent\_industry\_farming | 53 |
| solar\_installations | 54 |
| `play_Granite Wash` | 55 |
| total\_oil | 56 |
| percent\_age\_25\_44 | 57 |
| percent\_age\_65 | 58 |
| ethnic\_diversity | 59 |
| unemployment\_rate | 60 |
| social\_capital | 61 |
| percent\_mobility\_out\_mig | 62 |
