# Supplementary material for: Predicting firm creation in rural Texas: A multi-model machine learning approach to a complex policy problem
Source: PLoS One. 2023 Jun 23;18(6):e0287217. doi: 10.1371/journal.pone.0287217 (PMC10289456; doi:10.1371/journal.pone.0287217)
Supplement: Attachment — Submitted filename: Response to Reviewers.docx [file pone.0287217.s004.docx]

### Response to Reviewers

### Reviewer #1

This paper examines the factors driving the creation of firms in rural America. In doing so, the authors employ a Multi-Model Machine Learning Approach. The authors find that some factors that promote entrepreneurship may not be as predictive as socioeconomic ones. Moreover, the strength of specific industries predicts firm growth, as does the number of local banks. Finally, the authors provide some policy implications of their findings.

The authors have addressed all the comments that I raised before. They have done a great effort to include new machine learning methods. I believe the paper could be published in its current form.

*Thank you for this vote of confidence! And thank you for your earlier review; we do think this is a much stronger paper for it.*

### Reviewer #2

The main problem is the introduction part, of course which can be written in a variety of ways, but some important things still need to be clarified:

1) Research objectives. The paper asks a general question about "what can predict firm creation in rural America". But obviously the first paragraph does not address around the essence of this question (what's legitimacy of your research objective? from which aspect you will cut into this issue? what you will get and why it's important in theory or practice?... ). Promoting rural entrepreneurship is certainly important, but does it have anything to do with the question you raised in the first sentence? If so, what is the logic? If your logic is that the US government can intervene to promote rural entrepreneurship based on what machine learning predicts, that please gives us enough evidences that this logic can be implemented at the state level of the United States... Don't leave anything behind which may make reviewers and audiance confused and to guess.

*We thank Reviewer #2 for helping us sharpen our introduction overall. Specifically, first we have made revisions throughout the introduction to first set the stage for the why rural entrepreneurship is important and to identify the gaps in the literature. Before we get to the general question of what factors are predictive of firm creation in rural America, we have added some context on why this question is important. On page 3 paragraph 1, we added the following text:*

*“In the United States, the years following the Great Recession of the late 2000s is a tale of two recoveries. While urban areas saw slow, steady recovery, in the run-up to the 2016 election, some rural states had yet to return to their pre-Recession levels employment. Closing that gap means building the economies of rural areas; this, in turn, depends on higher rates of entrepreneurship and self-employment, which can help communities jumpstart and sustain economic growth, create jobs, and mitigate trade shocks more effectively than attracting large firms.*

*In the second half of the revised introduction, after we have more clearly identified existing gaps in research (detailed in response to comment #2 below), we added a new paragraph laying out our research objectives explicitly (p7). Here is that text, for ease of reviewing:*

*“This paper addresses each of those gaps in prior research, with three research objectives. First, we aim to provide an answer to the question of what factors predict firm creation in rural America. Second, we aim to demonstrate how those factors differ from urban America. Third, we aim to demonstrate how policy scholars and the policymakers they advise can deploy computational methods to complex policy problems without monocausal explanations.”*

*Therefore, the question “what can predict firm creation in rural America?” is introduced as an analytical entryway to address these gaps in the current literature. In addition to changes throughout the introduction section, we have made related changes to the abstract as well. The abstract now clearly lays out the research gaps we address and why addressing these gaps is important. The first paragraph of the revised abstract (page 2) is as follows:*

*“Rural and urban Americans has becoming increasingly divided, both politically and economically. Rural entrepreneurship can help communities catch back up by jumpstarting economic growth, creating jobs, and building resilience to economic shocks. However, less is known about firm creation in rural areas compared to urban areas. To that end, in this paper we ask: What factors predict firm creation in rural America? Our analysis, based on a comparative framework involving multiple machine learning modeling techniques, helps addresses three gaps in academic literature on rural firm creation. First, entrepreneurship research stretches across disciplines, often using econometric methods to identify the effect of a specific variable, rather than comparing the predictive importance of multiple variables. Second, research on firm creation centers on high-tech, urban firms. Third, modern machine learning techniques have not yet been applied in an integrated way to address rural entrepreneurship, a complex economic and policy problem that defies simple, monocausal claims. In this paper, we apply four machine learning methods (subset selection, lasso, random forest, and extreme gradient boosting) to a novel dataset to examine what social and economic factors are predictive of firm growth in rural Texas counties from 2008-2018.”*

*We thank the reviewer again for strengthening our articulation of the research objectives and gaps.*

2) Research gap. Not recommend to demonstrate the existing research by table here, it's not enough to just throw out some evidences, stating logic and viewpoint is your job and not homework of audience. In the introduction part, our concern have not reached the specific determinants and varialbles level yet , but more concerned your comment about the progress of research at the general level. This will show your familiarity with the field, your understanding of the problem, and the legitimacy of your following research gap summary. Furthermore, your summary of the research gap is not logical enough to connect the research objectives and existing research.

*We agree with the reviewer that the research gaps were not clearly articulated. While we did identify the gaps in current literature, the prior introduction framed these gaps as obstacles to application by policymakers, rather than gaps in existing research.*

*We reworked the introduction to make clear the three research gaps this paper fills. We derive these gaps based on our analysis of the previous theoretical and empirical studies as summarized in Tables 1 and 2. While we do not delve into explaining each of these studies in detail, we observe that the research on rural firm creation is theoretically disconnected, and most of the studies do not place an explicit focus on entrepreneurship dynamics in rural counties. Accordingly, we have summarized three research gaps that we address in this paper. These research gaps are detailed on pages 5 and 7 in the revised manuscript.*

*The three research gaps are –*

- *first, the absence of research pulling together what research does exist into a single study across disciplines (page 5 para 2);*
- *second, the relative dearth of research on rural entrepreneurship versus urban entrepreneurship (page 5 para 3); and*
- *third, the absence of the application of machine learning methods in rural entrepreneurship research (page 7 para 1).*

3) Margin contribution. Obviously I didn't find it in introduction part. Explain what you have done to fill the research gap and what is the incremental academic value of doing so.

*We thank the reviewer for pointing out this omission. At the end of our updated introduction, we have included a few paragraphs summarizing the research objectives, what we have done to fill these research gaps, and provide a roadmap of the rest of the paper, making clear to whom this research is relevant. We save most of our discussion of contribution for the discussion section. For ease of reviewing, the new paragraphs on page 8 in the revised manuscript are reproduced below:*

*“This paper addresses each of those gaps in prior research, with three research objectives. First, we aim to provide an answer to the question of what factors predict firm creation in rural America. Second, we aim to demonstrate how those factors differ from urban America. Third, we aim to demonstrate how policy scholars and the policymakers they advise can deploy computational methods to complex policy problems without monocausal explanations.*

*To accomplish this, we pull together variables identified by multiple disparate strands of rural entrepreneurship research into machine learning models to identify what factors predict the of firm growth in 254 Texas counties across ten years. We also identify how the factors that predict rural entrepreneurship differ from the factors that predict urban entrepreneurship, both in prior research and in our own models, as we expected.*

*In the next section of this paper, we describe what data we collected, and how. We then describe each of the four machine learning models we deployed, why we chose them, and how we combine the results of those models into a single output that policymakers can make sense of. We then share the results of each individual model of rural firm creation, the combined results, and a comparison of those results to our urban firm creation models.*

*In the discussion, we interpret those results, including their implications for rural entrepreneurship scholars and policymakers alike. We then consider how policy scholars and practitioners might apply this approach to other complex policy issues.”*

Generally speaking, the narrative in the introduction part is insufficient and appropriate, which hinders the audience's jugement of the importance and innovation about this research. Don't let reviewers and audiance to fill the blanc of (why, what, how and whether good you have done) for you, they don't like it.

*We thank the reviewer again for the detailed review suggesting ways to improve the introduction section of this manuscript. We believe the updated introduction more clearly lays out the “why, what, how” of this research. Clearer articulation of the research gaps and our approach to addressing them better sets up the audience for the rest of the manuscript. Together with the methodological updates made in the previous round of review, we believe the manuscript has strengthened both in terms of its clarity and its robustness.*
